# Male‐territory‐visiting polygamy in the sand‐dwelling goby *Fusigobius inframaculatus* (Gobiidae) inhabiting reef caves

**DOI:** 10.1111/jfb.70422

**Published:** 2026-03-24

**Authors:** Ryoga Seiwa, Yoichi Sakai

**Affiliations:** ^1^ Graduate School of Integrated Sciences for Life Hiroshima University Hiroshima Japan

**Keywords:** field observation, *Fusigobius*, mating system, reproductive biology, sexual dimorphism

## Abstract

We conducted an observational field survey of the innerspotted sandgoby *Fusigobius inframaculatus* on reefs of Kuchierabu‐jima Island, southern Japan, to investigate its mating system. Individuals of the goby maintained home ranges on sandy bottoms within rocky caves or beneath overhangs that served as shelters. Of 34 identified individuals, 32% disappeared during the 4‐month reproductive season, and nearly all had disappeared by the next reproductive season, indicating a high mortality rate. Territorial males constructed spawning nests on the ceilings of these cavities and mated with multiple females. The mating groups were isolated from one another and intergroup movements were rare, suggesting restricted movement under a strong predation pressure. Although facultative harem polygyny was observed in some local groups, most females visited male territories from outside for spawning and often changed mates. Based on the segregated spatial relationships of home ranges between sexes and unstable mating relationships, the mating system was characterized as male‐territory‐visiting polygamy. The goby exhibited sexual dimorphism in body size, with males being the largest individuals within local groups, and exhibited subtle and transient coloration changes in males during courtship, which may function as female choice traits. Our results suggest that the polygamous mating system in *F*. *inframaculatus* is maintained even under constraints of mate availability due to limited mobility within the narrow sheltering habitat at the boundary between sandy bottoms and rocky substrates.

## 1 INTRODUCTION

The spatial distribution of individuals and the economics of defending resources and mates are key factors determining the mating systems of animals (Emlen & Oring, 1977). When resources are unevenly distributed or spatially clumped, resource‐defence polygyny becomes advantageous, as males can indirectly gain access to multiple females by defending these resources. A typical form of resource defence polygyny in reef fishes is male‐territory‐visiting (MTV) polygamy, in which females are usually located outside male mating territories, resulting in unstable mating relationships (Kuwamura, 1997; Sakai, 2023). In contrast, harem polygyny is another common mating system in reef fishes, in which multiple females cohabit within a single male's territory, resulting in stable mating relationships (Kuwamura, 1997; Sakai, 2023). Resource defence and/or female defence territoriality are suggested to underlie haremic mating systems in various reef fishes (Gladstone, 1987; Seki et al., 2009; Sunobe & Nakazono, 1990).

Many gobiid fishes are habitat specialists, utilizing diverse environments such as rocky substrates, sandy bottoms, caves and corals (Herler et al., 2011). Generally, gobies spawn demersal eggs in spawning nests and males provide parental care (Blumer, 1979). MTV polygamy has been reported in four gobiid species: *Fusigobius neophytus* (Günther, 1877), *Asterropteryx semipunctata* Rüppell, 1830 and *Hazeus ammophilus* Allen & Erdmann, 2021 in sandy habitats, and *Bathygobius fuscus* (Rüppell, 1830) in rocky habitats (Kawase & Tsuhako, 2026; Manabe et al., 2009; Taru et al., 2002; Tsuboi & Sakai, 2016). Harem polygyny in gobies has been reported mainly in species of the genus *Trimma*, which inhabit caves and holes in rocky reefs (Fukuda et al., 2017; Fukuda & Sunobe, 2020; Sunobe & Nakazono, 1990), and is considered to be driven by resource defence territoriality (Sunobe & Nakazono, 1990).

Protogyny (female‐to‐male sex change) and bidirectional sex changes have been reported in multiple gobiid genera (Cole, 2010; Kuwamura et al., 2020, 2023). The evolution of sex change is explained by the size‐advantage (SA) model (Warner et al., 1975). In polygynous or polygamous mating systems in fishes involving MTV polygamy and harem polygyny, where females prefer larger males and/or large males monopolize mates, male reproductive success increases exponentially with body size, whereas female reproductive success increases linearly. Under these conditions, protogyny is adaptive because individuals gain higher reproductive success by functioning first as females at smaller sizes and later as males after attaining a larger body size. Because sex change is closely linked to mating systems, the mating systems of many hermaphroditic gobiid species inhabiting corals (e.g. genera *Paragobiodon* and *Gobiodon*; Kuwamura et al., 1993; Munday et al., 1998; Nakashima et al., 1996) and rocky environments (e.g. genera *Trimma* and *Priolepis*; Fukuda et al., 2017; Fukuda & Sunobe, 2020; Sunobe & Nakazono, 1990, 1999) have been investigated through detailed field observations. However, detailed information on the mating systems of sand‐dwelling hermaphroditic gobies, including *Fusigobius*, remains poorly understood under natural conditions.

The genus *Fusigobius* is one of the hermaphroditic lineages within Gobiidae and has been suggested to exhibit protogyny (Burns & Cole, 2017; Cole, 2010). This genus comprises small benthic gobies distributed across subtropical to tropical regions of the Indo‐Pacific Ocean, inhabiting sandy and rubble bottoms adjacent to rocky substrates (Nakabo, 2013; Randall, 2001). Among them, *F. neophytus* is the only species for which MTV polygamy has been reported, based on detailed field surveys (Tsuboi & Sakai, 2016). The congeneric *Fusigobius inframaculatus* (Randall, 1994), the focal species of this study, is morphologically characterized by elongated filamentous dorsal fin spines (Randall, 1994). This species is known to inhabit sandy bottoms within rocky caves and beneath rocky overhangs, and exhibits different habitat use compared with *F. neophytus*, which inhabits open sandy areas.

Despite these differences in habitat use, the reproductive behaviour and mating system of *F. inframaculatus* remain undescribed. Does this species also exhibit a similar mating system, despite its distinct habitat use compared with its congener *F. neophytus*? This study aimed to clarify the ecological and social mechanisms underlying the mating system of *F. inframaculatus* under natural conditions through detailed field surveys of spatial organization and mating relationships. By describing the ecological characteristics, resource‐defence territoriality and mating behaviours of *F. inframaculatus*, we discuss how habitat structure and ecological constraints shape the mating system of this species.

## 2 MATERIALS AND METHODS

### 2.1 Field observation

**FIGURE 1:**
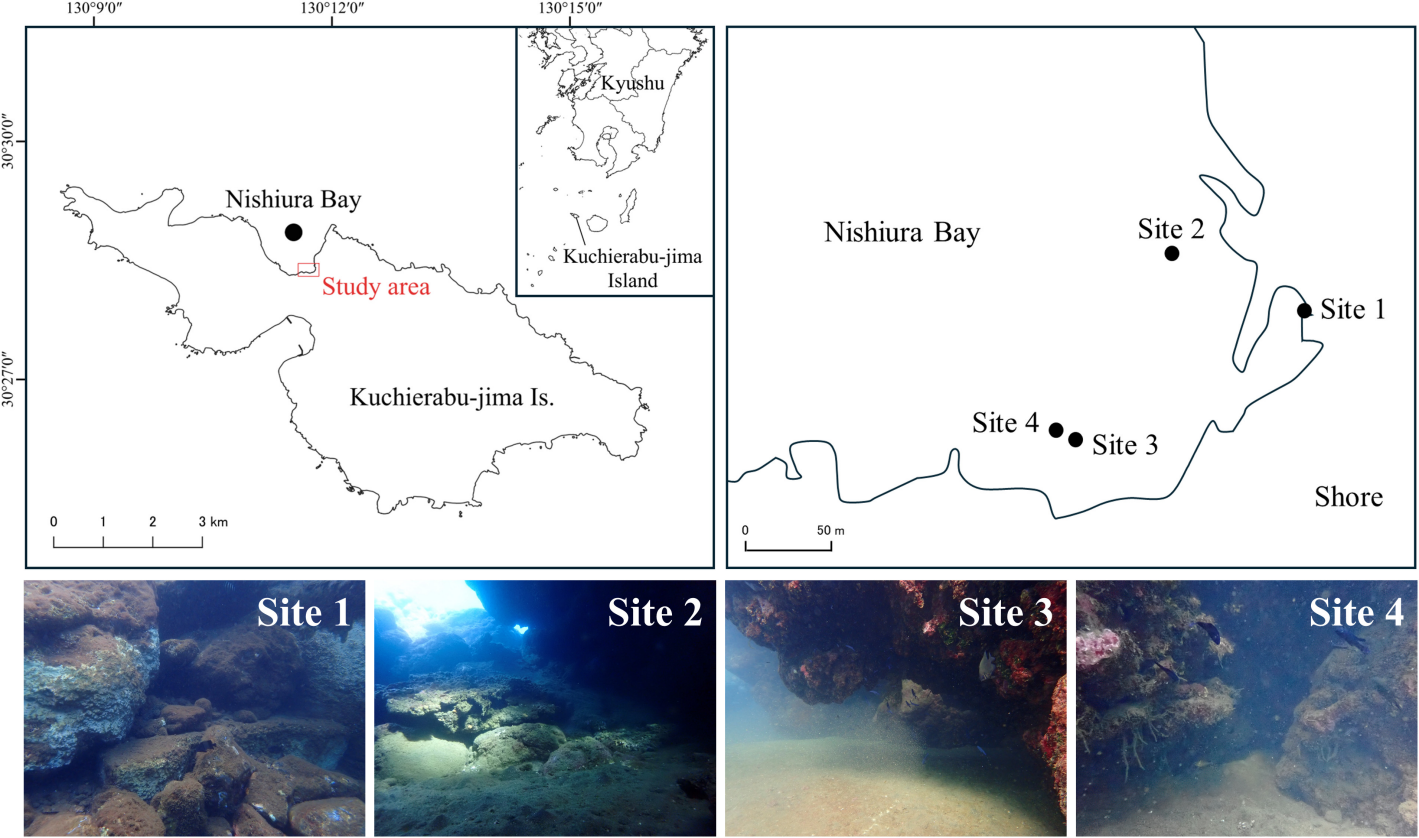
Map of the study area of *Fusigobius inframaculatus* on the reef of Kuchierabu‐jima Island, southern Japan. The four study sites are indicated (water depth): Site 1 (2.7 m), Site 2 (7.1 m), Site 3 (6.0 m) and Site 4 (6.7 m).

We conducted field observations of a population of *F. inframaculatus* in Nishiura Bay, Kuchierabu‐jima Island (30°28′30″N, 130°11′30″E), southern Japan (Figure 1). *F. inframaculatus* is commonly found along the coast of Kuchierabu‐jima Island on sandy substrates within caves and beneath overhanging rocks (Kimura et al., 2017; Figure 1). Underwater observations were made using SCUBA from early June to early October 2024, when the water temperature ranged from 24.1 to 31.2°C. The home range of this species was limited to shaded areas within caves or under overhangs, and social interactions were observed only among individuals within these areas (see Results). Therefore, each topographically contiguous shaded area within caves or beneath overhangs was considered a single study site. Four study sites (2.7–7.1 m depth) were established (Figure 1): Site 1 (2.5 × 2.5 m), Site 2 (12.0 × 8.5 m), Site 3 (13.3 × 12.6 m) and Site 4 (4.4 × 3.2 m). The distance among sites was a median of 128 m (range 13–163 m). Site topography was surveyed using measuring tapes, an underwater digital camera (Tough TG‐5; Olympus), and a compass, and maps of each site were drawn on waterproof sheets.

At the start of the study (early June 2024), all visible individuals were captured using a hand net and 1% clove oil solution. Captured fish were transported to a local laboratory and the total length (TL) was measured to the nearest 1 mm using callipers. Sex was determined by genital papilla morphology: males possess long, posteriorly tapered papillae, whereas females have bulbous papillae with several processes at the opening (Randall, 1994). To distinguish individuals, we subcutaneously injected a visible fluorescent elastomer tag (Northwest Marine Technology Inc.) into the body. Marked fish were released at their capture locations within 24 h, after confirming normal swimming behaviour on a holding tank. Unmarked individuals found later were measured and marked using the same protocols. Individuals larger than 40 mm TL were considered reproductively mature (see Results), while smaller individuals were excluded from behavioural observations. During the study, seven males and 30 females were individually identified.

Census surveys were conducted at all sites almost daily (0630–1100) between 10 June and 21 September 2024. Social interactions occurred only among individuals within the same site, therefore fish at each site were regarded as members of the local social groups. Survival rates during the reproductive season (June–September 2024) were analysed and compared between sexes. Additional census surveys were conducted from late June to early July 2025 to estimate survival rates for the following spawning season.

**FIGURE 2:**
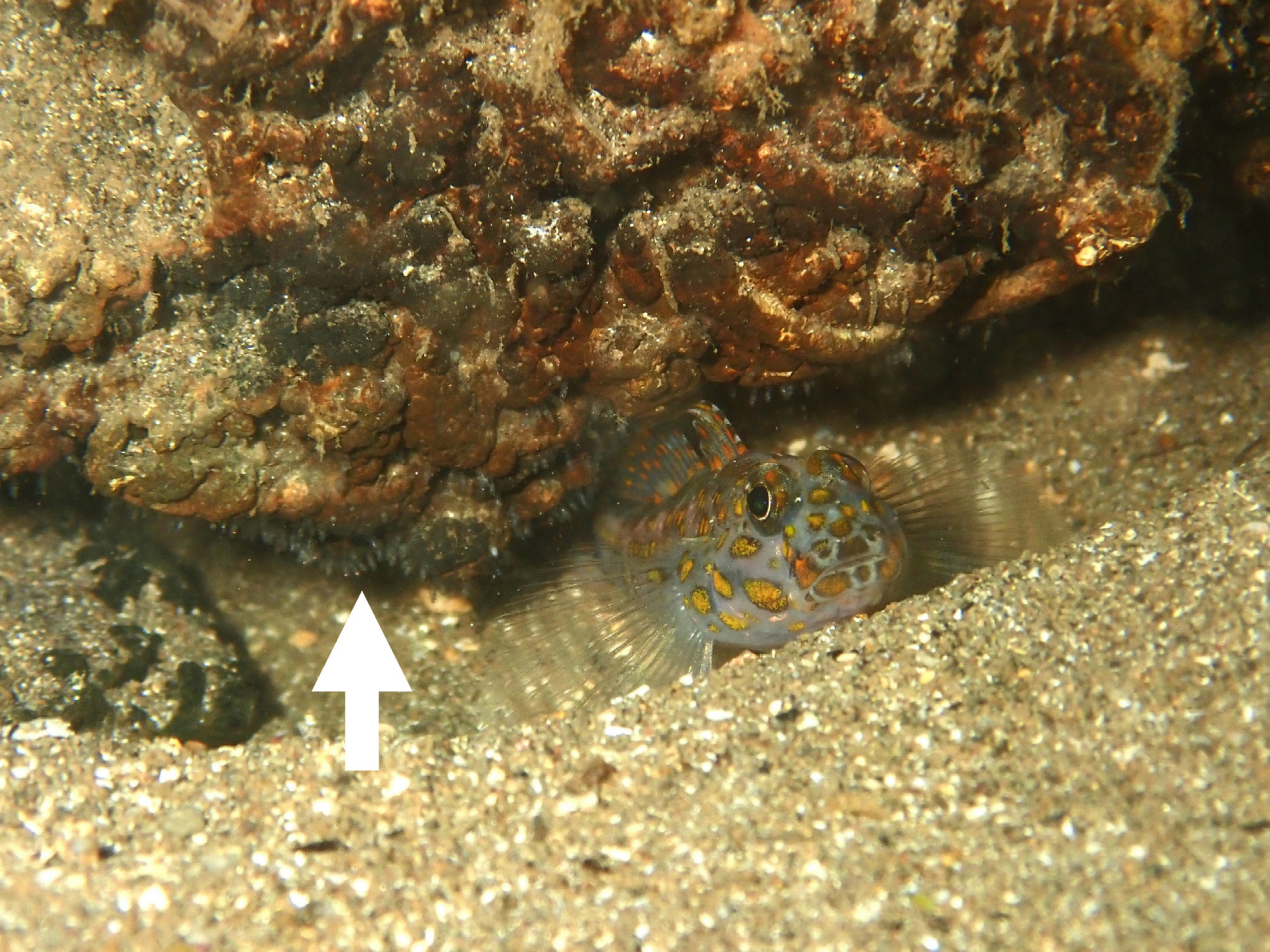
A nest‐holding male *Fusigobius inframaculatus* guarding eggs in a spawning nest. Eggs were laid on the ceiling of the cavity. The white arrow indicates eggs.

Males of *F. inframaculatus* establish mating nests and females visit these nests for spawning, as observed in the congeneric *F. neophytus* (Tsuboi & Sakai, 2016). We recorded the identity of each mating pair and analysed pair stability using continuous census data for periods without male disappearance or weather interruptions: Site 1, 10 August–26 August 2024 (17 days); Site 2, 6 August–26 August 2024 (21 days); Site 3, 29 July–26 August 2024 (29 days); Site 4, 6 July–26 August 2024 (51 days). Because nests were located on the ceilings of narrow cavities (Figure 2), it was difficult to count the eggs, therefore reproductive success was estimated from the number of observed spawning events over 78 days (10 June–26 August 2024) without interruptions from bad weather. In addition, five spawning events were recorded using underwater video cameras (GoPro HERO 6 and HERO 8; GoPro Inc.).

To analyse spatial organization, we recorded individual movement paths on the site maps during repeated 10 min focal observations conducted between 0730 and 1830. Each individual's home range was defined as the area enclosed by the most peripheral path recorded for three 10 min observations. For males, movement paths were recorded only when they were not engaged in egg care. Intraspecific social interactions were also recorded during these observations. When spawning behaviour was observed before or after recording movement paths, those data were excluded to eliminate the influence of spawning migration. Home range areas (m^2^) were analysed for Site 3 (12 individuals) and Site 4 (three individuals) using Image J (Schneider et al., 2012). Measurements at Sites 1 and 2 were not obtained because of the complex and narrow reef topography. Home range surveys were conducted from 21 to 27 July 2024 at Site 3, and 21 June to 5 July 2024 at Site 4. The home range overlap rate between individuals was calculated as 100 (%) × overlapping area within the home range of the smaller individual (m^2^)/home range area of the larger individual (m^2^). The midpoint of the longest side of each female's home range was designated as the centre of the home range and the distance from this point to the spawning nest was measured.

### 2.2 Video analysis and specimen sampling to confirm sexual dimorphism

From five recorded videos of spawning behaviour, we identified the individuals present and determined their sex. For each individual in each video, the presence or absence of transient courtship coloration (see Results) was recorded.

In April 2025, five males and eight females of *F*. *inframaculatus* were captured outside the observation area in Nishiura Bay using a hand net and 1% clove oil to analyse morphology. After capture, the specimens were euthanized immediately with an overdose of anaesthetic and fixed in 10% formalin. During courtship displays and social interactions, the goby often expanded its dorsal and caudal fins (see Results), therefore TL, body weight (BW), caudal‐fin length (CFL), elongated‐dorsal‐fin‐ray length (EDFRL) and anal‐fin‐ray length (AFRL) were measured to 0.01 mm using a digital calliper. Sex was determined based on the morphology of the genital papilla.

### 2.3 Statistical analysis

All statistical analyses were conducted in R version 4.2.2 (R Core Team, 2024) with a significance level of 0.05. Nonparametric tests were used because most datasets did not meet the assumptions of the parametric tests. The Mann–Whitney *U* test was applied for two‐group comparisons and the Kruskal–Wallis test for multiple groups. Fisher's exact test was used for proportion comparisons, Spearman's rank test for correlation analyses and the binomial proportion test (expected probability = 0.5) for ratio analyses. Analysis of covariance (ANCOVA) was used to compare the regression lines.

To analyse the factors affecting reproductive success, we used generalized linear models (GLMs) with a negative binomial distribution, as overdispersion was detected in Poisson models. Negative binomial GLMs were constructed using the glm.nb function in the MASS package. The reproductive success of males or females was used as the response variable, with body size as the explanatory variable. Because some individuals appeared or disappeared during the 78 days of observation, the observation period for each individual (from the first to the last sighting) was included as an offset. Additionally, we constructed a negative binomial GLM to compare reproductive success between the sexes, with sex as the explanatory variable. Individuals that underwent sex change or non‐territorial males were excluded from these analyses due to small sample sizes.

## 3 RESULTS

### 3.1 Group composition at the four study sites

**FIGURE 3:**
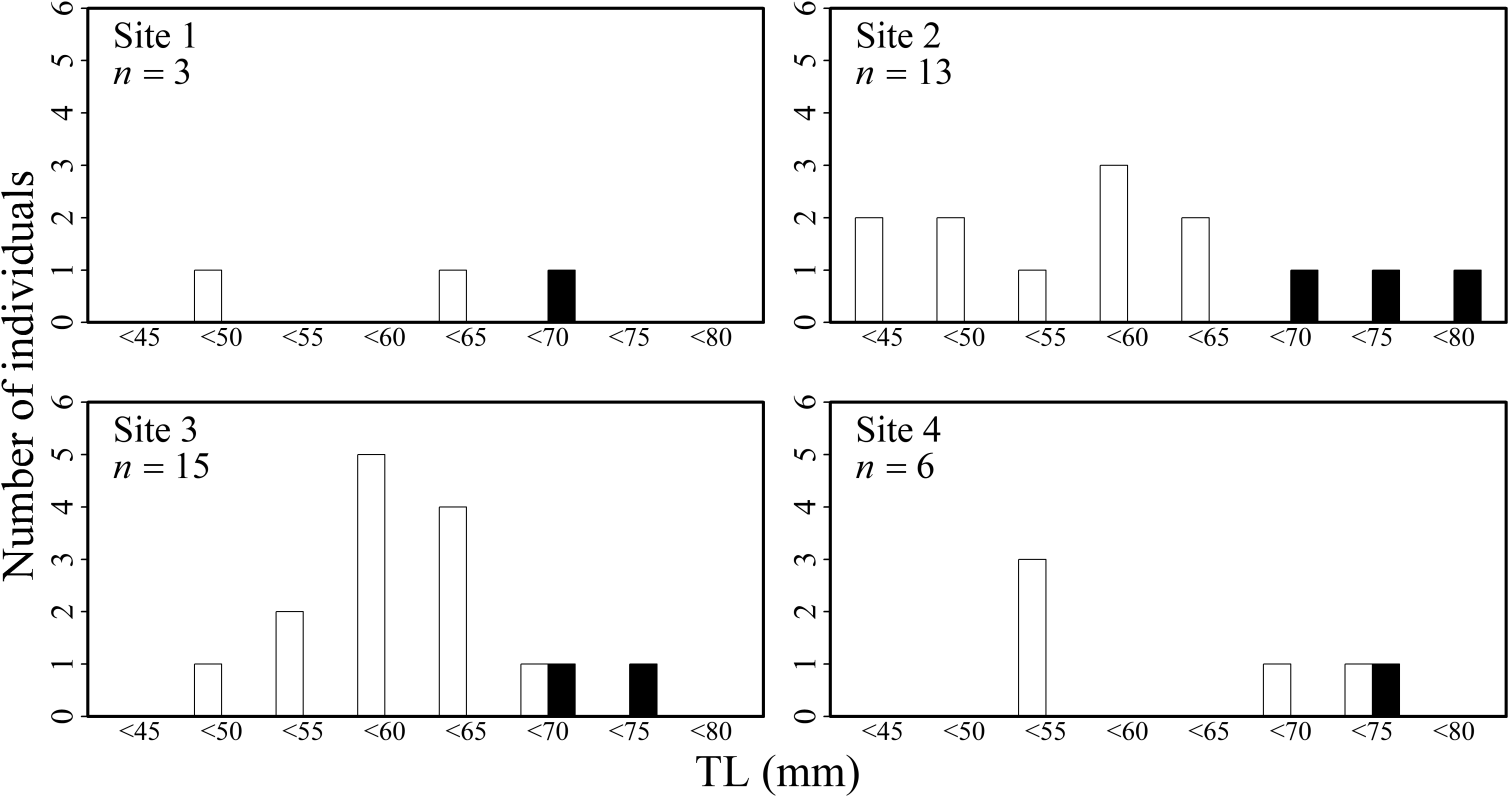
Size composition of mating groups of *Fusigobius inframaculatus* on the reef of Kuchierabu‐jima Island. Open bars, females; solid bars, males. TL, total length.

Each study site contained three to 15 individuals (median 10; Figure 3). At each site, males were consistently the largest individuals, ranked from the largest to the third largest in each group, and no females exceeded the size of males (Figure 3 and Table S1). At Sites 2 and 3, males were significantly larger than females (Mann–Whitney *U* test; Site 2, *U* = 30, *p* < 0.05; Site 3, *U* = 26, *p* < 0.05; Table S1). No significant differences in female TL were found among the four sites (Kruskal Wallis test, *df* = 3, χ^2^ = 2.7, *p* = 0.4; Table S1).

### 3.2 Spawning behaviour

Males constructed spawning nests on the upper surfaces of cavities between sandy and rocky substrates (Figure 2). Males excavated sand using their caudal fins during the nest construction. Spawning nest construction began between 6 days before and the day of female spawning (median 0 days; *n* = 37), with males intermittently removing sand until the spawning occurred. No significant differences were found among sites in the interval between nest construction and spawning (Kruskal–Wallis test, *df* = 3, χ^2^ = 2.7, *p* = 0.4).

A total of 47 spawning events were observed during the study period. When females approached male nests, males performed courtship displays by swimming around the female with their fins fully spread (Video S1). During courtship, the males exhibited blue‐banded markings along the edges of their dorsal and anal fins (Video S1). Females entered the nest to lay eggs in a single layer on the upper surface (Video S1). Immediately thereafter, the males directly smeared sperm over the laid eggs. During spawning events, the males alternated between courtship displays and sperm smearing. In addition, males frequently removed sand from their nests during spawning events. After spawning, the females immediately returned to their home ranges. Courtship and spawning behaviours did not differ among the sites. No female mated with more than one male on a single day. The median interval between spawning events for each female was 13 days (range 6–44 days, *n* = 32).

Eggs were cared for exclusively by males, who fanned them with pectoral fins and removed sand using their caudal fins. Egg care lasted 2–8 days (median 5 days, *n* = 37), with no site differences (Kruskal–Wallis test, *df* = 3, χ^2^ = 1.0, *p* = 0.8). After egg care, nests were covered with sand; males rebuilt nests after 1–9 days later (median 3 days, *n* = 34), with no site differences (Kruskal–Wallis test, *df* = 3, χ^2^ = 4.7, *p* = 0.2). Nests were reused repeatedly (94%, *n* = 34). Each male mated with females 1–12 days after completing egg care (median 3 days, *n* = 25), with no site differences (Kruskal–Wallis test, *df* = 3, χ^2^ = 4.65, *p* = 0.2).

Of the seven males, six maintained spawning nests (i.e. nest‐holding males; Tsuboi & Sakai, 2016), whereas only one male (M18, 66 mm TL, Site 2) did not maintain a spawning nest (i.e. floating male; Tsuboi & Sakai, 2016). At the time of the floating male's first appearance at Site 2, a larger male (M2, 73 mm TL) had already established a nest and spawned with females. M18 disappeared on 29 June 2024 (Table S1), 18 days after its first observation, without ever maintaining a nest. Sneaking behaviour was not observed in M18.

### 3.3 Mate fidelity and reproductive success

**FIGURE 4:**
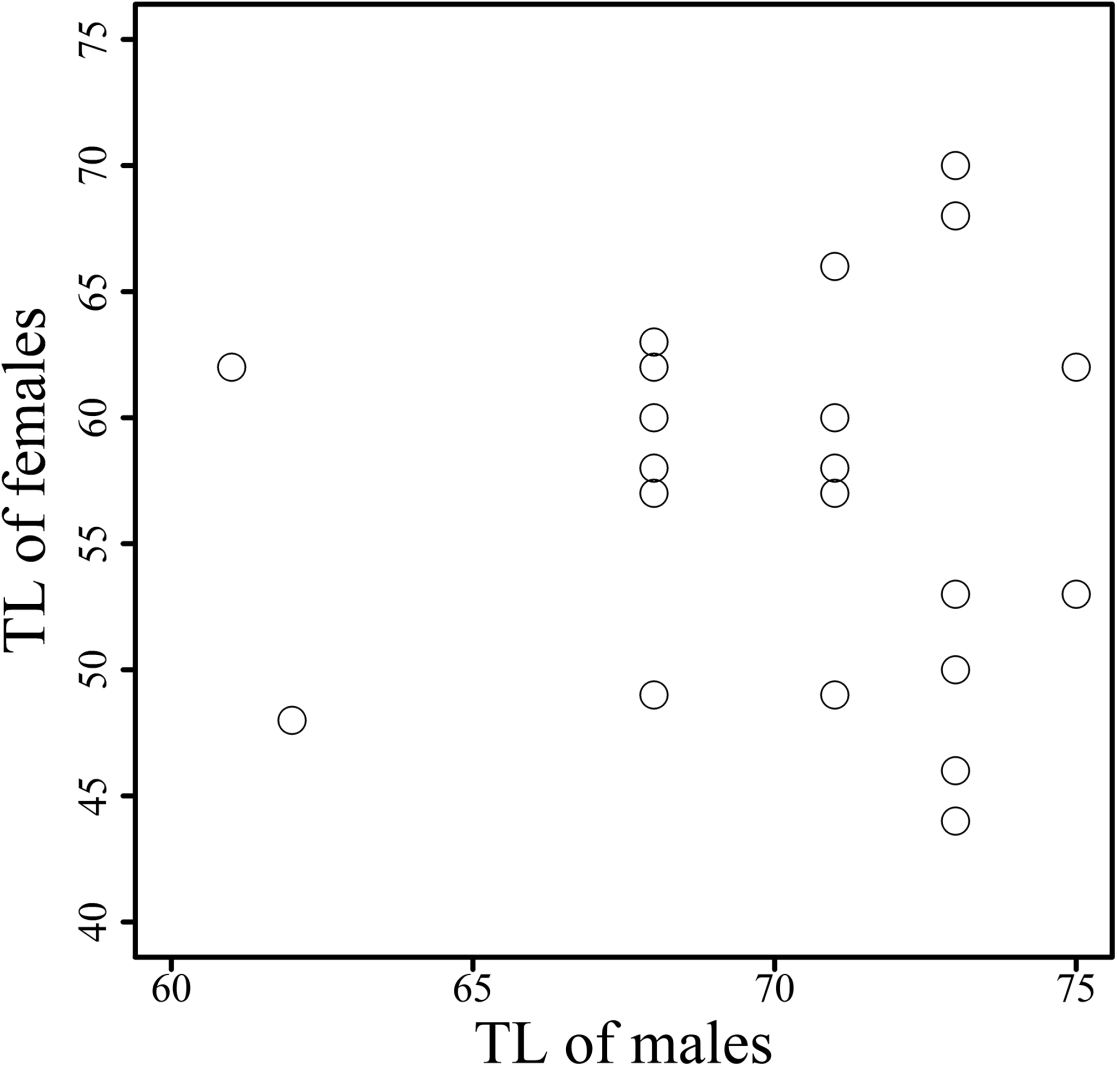
Relationship between total length (TL, mm) of males and female in the mating pairs of *Fusigobius inframaculatus*.

Males spawned with one to four females per day (median 1, *n* = 47), over 1–3 consecutive days. During a single egg‐caring period, males cared for eggs from one to four females (median 1, *n* = 36). There was no significant correlation between the TL of the mating pairs (Spearman's rank correlation coefficient, *r*_*s*_ = −0.08, *p* = 0.7, *n* = 23), indicating no evidence of size‐assortative pairing (Figure 4). No significant correlation between female TL and the number of mates was detected (Spearman's rank correlation coefficient, *r*_s_ = −0.09, *p* = 0.8, *n* = 10; Table 2).

At Site 2, one female (F29) changed mates between two males (Table 1a). Among the five females that spawned multiple times at Site 3, three (F9, F17 and F19) changed their mating partners within the group (Table 1b). Overall, of the six females observed spawning multiple times in these groups, four (67%) exhibited mate change (Table 1). In contrast, at Site 4, two females (F22 and F25) repeatedly spawned with the only male (M11) in the group during the 51‐day consecutive survey (Table 2), a pattern resembling harem polygyny. Although multiple spawning events were not observed at Site 1 during the 17‐day consecutive survey, one male (M1, previously F1) spawned with a cohabiting female (F31).

**TABLE 1:** Mating relationships in *Fusigobius inframaculatus* at Sites 2 (**a**) and 3 (**b**) during the continuous 21‐day (August 6 to August 26, 2024) and continuous 29‐day (July 29 to August 26, 2024) surveys, respectively.

(a) Site 2									
	Males ID	Females ID						Total spawning	Total mates
		F29 (62)	F5 (58)	F33 (57)	F6 (53)	F34 (45)	F16(44)		
	M32 (75)	1						1	1
	M24 (61)	1						1	1
Total spawning		2	0	0	0	0	0		
Total mates		2	0	0	0	0	0		

(b) Site 3													
	Males ID	Females ID										Total spawning	Total mates
		F13 (66)	F23 (63)	F21 (62)	F17 (60)	F19 (58)	F26 (59)	F9 (57)	F30 (57)	F12 (51)	F14 (49)		
	M8 (71)	2			1	1		1	1	1		7	6
	M27 (68)		1	2	1	2		2			1	9	6
Total spawning		2	1	2	2	3	0	3	1	1	1		
Total mates		1	1	1	2	2	0	2	1	1	1		

*Note*: The number of spawning events for each pair is shown. Individual codes indicate sex (M male, F female), with total length (TL, mm) in parentheses.

**TABLE 2:** Mating relationships of *Fusigobius inframaculatus* at Site 4 during the continous 52 ‐ day (6 July to 26 August 2024) surveys.

	Male ID	Female ID		Total spawning	Total mates
		F22 (70)	F25 (50)		
	M11 (71)	6	2	8	2
Total spawning		6	2		
Total mates		1	1		

*Note*: The number of spawning events for each pair is shown. Individual codes indicate sex (M, male; F, female), with total length (TL, mm) in parentheses.

**FIGURE 5:**
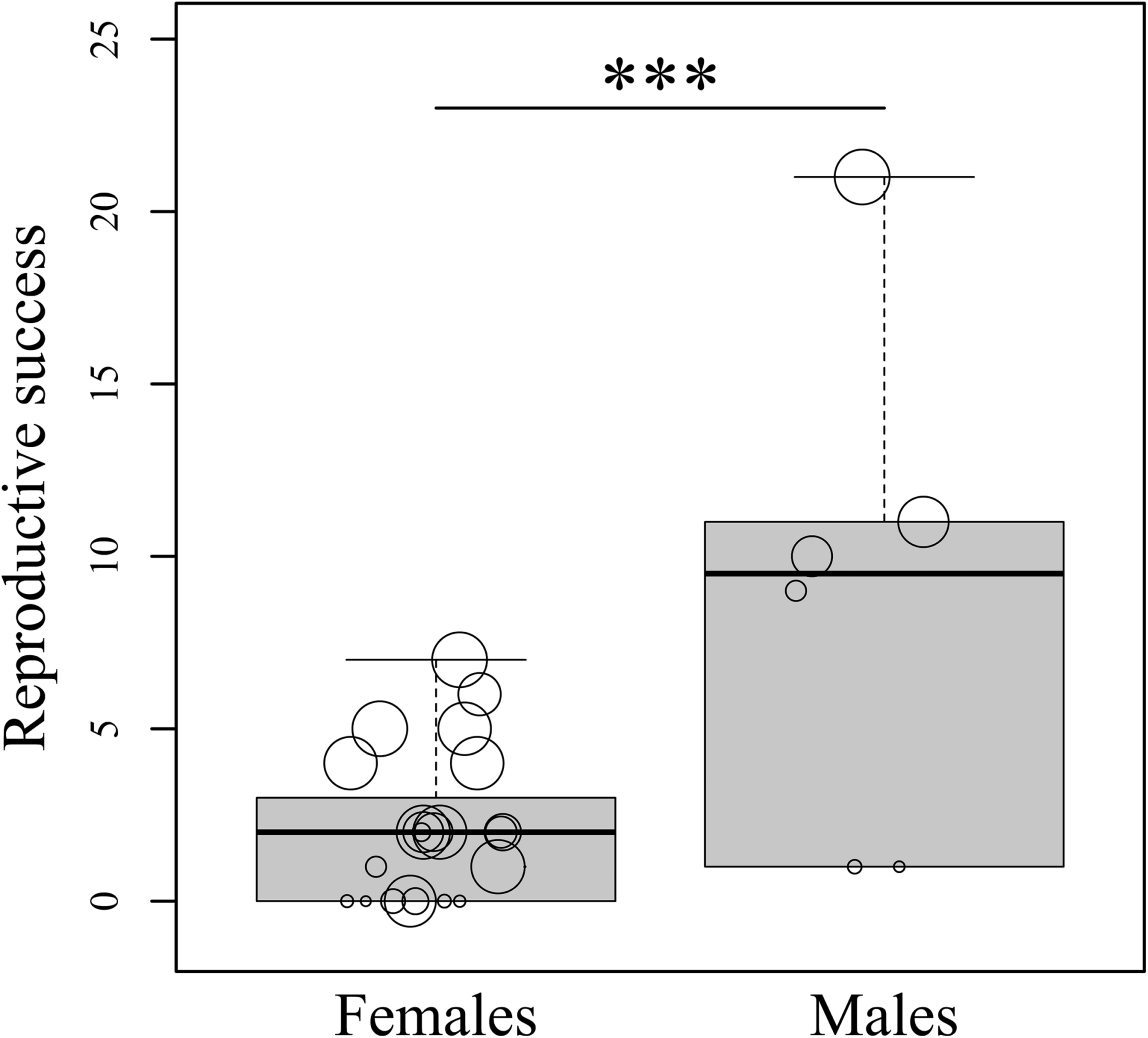
Reproductive success of females (*n* = 22) and males (*n* = 6) of *Fusigobius inframaculatus*. Statistical results are shown (negative binomial GLM, ***p < 0.001). The plot size indicates the number of survey days for each individual.

**FIGURE 6:**
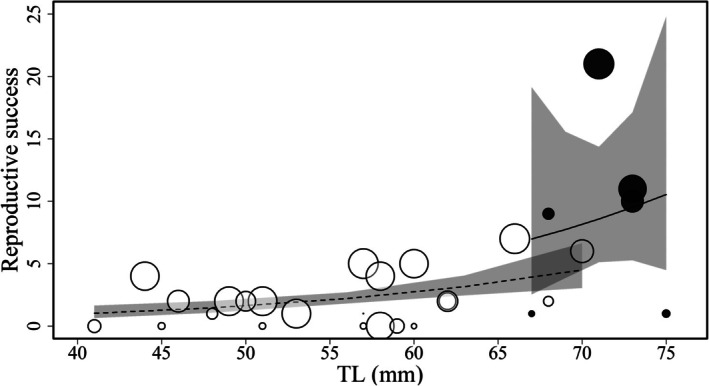
Relationship between reproductive success and total length (TL, mm) in females (*n* = 22; *white dots*) and males (*n* = 6; *black dots*) of *Fusigobius inframaculatus*. Dashed line represents the negative binomial generalized linear model (GLM) for females, with body size as the explanatory variable; *Solid line* represents that for males. *Shaded area* indicates the 95% confidence interval. *Plot size* indicates the number of observation days for each individual.

Male reproductive success was significantly higher than that of females (negative binomial GLM, estimate ± standard error [SE] = 1.45 ± 0.21, *df* = 1, *χ* ^2^ = 42.3, *p* < 0.001; males, *n* = 6; females, *n* = 23; Figure 5). In females, TL had a significantly positive effect on reproductive success (negative binomial GLM, estimate ± SE = 0.05 ± 0.01, *df* = 1, *χ* ^2^ = 15.0, *p* < 0.001, *n* = 23; Figure 6). In males, TL had no significant effect (negative binomial GLM, estimate ± SE = 0.01 ± 0.06, *df* = 1, *χ* ^2^ = 0.02, *p* = 0.84, *n* = 6; Figure 6).

### 3.4 Home range distribution and overlap patterns

**FIGURE 7:**
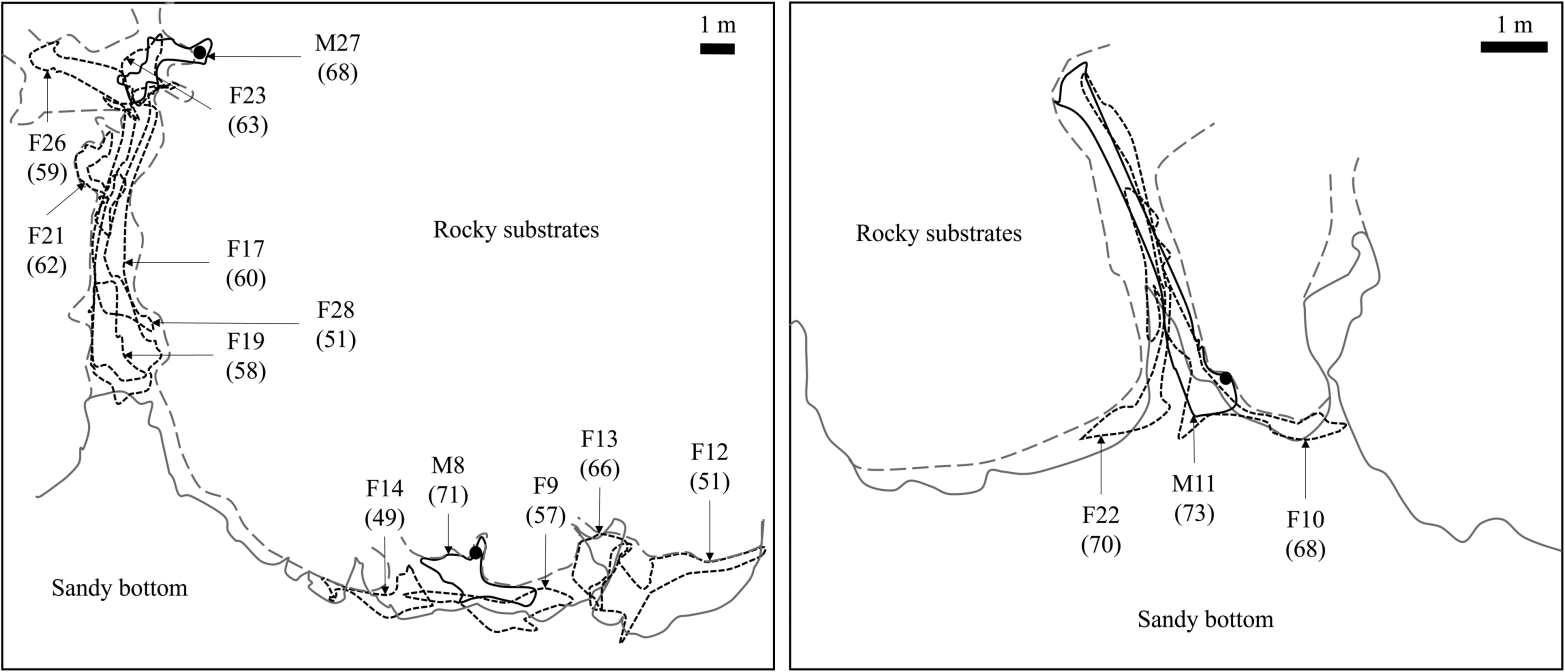
Home range distributions of *Fusigobius inframaculatus* at Site 3 (left) and Site 4 (right). Individual codes indicate sex (M, male; F, female), with total length (TL, mm) in parentheses. Black solid lines, male home ranges; black dotted lines, female home ranges; grey solid lines, shape of rocky substrates; grey dotted lines, outline of caves and undersides of overhangs; black dots, spawning nests.

During the home range survey, Site 3 comprised 12 individuals (two males and 10 females) and Site 4 comprised three individuals (one male and two females; Figure 7). Home ranges were confined to sandy bottoms (median 100%, range 85.7%–100%; Figure 7) and individuals rarely ascended vertical rock walls (Figure 1). All individuals primarily occupied shaded areas within caves or under overhanging rocks (median 99.3% of the home range, range 58.3%–100%; Figure 7).

At Site 3, the two males maintained non‐overlapping home ranges (overlap ratio 0.0%, *n* = 2; Figure 7), each including a spawning nest (Figure 7). Similarly, at Site 2, two territorial males maintained distinct spawning nests. At Site 4, a single territorial male maintained one spawning nest (Figure 7) and the same pattern was observed at Site 1. Spawning nests were constructed within the male home ranges (Figure 7).

At Site 3, two males occupied home ranges of 2.1 and 1.7 m^2^ (*n* = 2), while the median size of the female home ranges was 2.1 m^2^ (range 1.4–8.8 m^2^, *n* = 10). Half of the females (*n* = 5; Figure 7) overlapped with males, though the home range overlap rate was generally low (median 5.2% of the female home range, range 0.1%–59.2%, *n* = 5; Figure 7). Female home ranges partially overlapped with each other (median 19.7% of the female home range, range 4.5–99.0%, *n* = 10; Figure 7). No significant correlation was found between the differences in female TL and overlap rates (Spearman's rank correlation coefficient, *r*_s_ = 0.05, *p* = 0.8, *n* = 45). The median distance from male nests to female home ranges was 6.0 m (range 1.8–23.6 m, *n* = 11) and female TL was not correlated with the distance to spawning nests (Spearman's rank correlation coefficient, *r*_s_ = −0.29, *p* = 0.4, *n* = 12).

At Site 4, the single male had a home range of 1.9 m^2^ (*n* = 1), while the two females occupied home ranges of 1.1 and 1.7 m^2^ (*n* = 2). The male's home range overlapped with both females (overlap rate 56.4% and 13.1% for F10 and F22, respectively; Figure 7). Similar to Site 3, female–female overlap rates at Site 4 also were low (overlap ratios 8.2 and 13.1% of the F10 and F22 home ranges; Figure 7).

### 3.5 Intra‐ and inter‐specific interactions

When approached by conspecifics, *F. inframaculatus* performed aggressive display by spreading all fins and showing the body laterally (lateral display), observed in both sexes. Chasing and biting were also sometimes recorded. In male–female interactions (*n* = 15), the aggressors (i.e. males) were always larger than the recipient (i.e. females) (100%, binomial test, *p* < 0.001). Among females (*n* = 20), larger individuals were aggressors in most cases (95%, binomial test, *p* < 0.001, *n* = 20). Recipients often showed a submissive display by rapidly fluttering their caudal fins (*n* = 1 in male–female and *n* = 6 in female–female interactions). Aggressive interactions between territorial males were not observed.

Interspecific encounters were observed five times in this study. The carnivorous dottyback *Labracinus cyclophthalmus* (Müller & Troschel, 1849) chased *F. inframaculatus* (*n* = 2) and the piscivorous trumpetfish *Aulostomus chinensis* (Linnaeus, 1766) targeted *F. inframaculatus* (*n* = 1) as their prey. All targeted *F. inframaculatus* individuals sought shelter in cavities within the rocky substrate. In addition, nest‐holding males were observed using their caudal fins to throw sand at *L. cyclophthalmus* (*n* = 1) and the swimming crab *Thalamita* sp. (*n* = 1) on encounter.

### 3.6 Disappearance rates

During the behavioural observation period (July–September 2024), 43% of males (*n* = 3) and 27% of females (*n* = 8) disappeared, yielding an overall disappearance rate of 32% (excluding sex change individuals, *n* = 3) (Table S1). Disappearance rates did not significantly differ between the sexes (Fisher's exact test, *p* = 0.7). Of the 26 individuals that survived until September 2024, 88% (*n* = 23) disappeared before the following reproductive season in July 2025 (Table S1). The remaining three individuals were found at the same study sites in 2025.

### 3.7 Sexual difference in morphology

In the video recordings of spawning behaviour (*n* = 5), all males consistently exhibited blue‐banded markings along their caudal and anal fins (Video S1). In contrast, such markings were not observed on the fins of females during courtship and spawning (Video S1).

In the sampled specimens, the total length (TL) of males was significantly larger than that of females (Mann–Whitney *U* test, *U* = 34, *p* < 0.05; males, median 70.5 mm TL, range 46.1–75.4 mm TL, *n* = 5; females, median 47.4 mm TL, range 37.7–47.4 mm TL, *n* = 8). The body weight (BW) of males was also significantly higher than that of females (Mann–Whitney *U* test, *U* = 34, *p* < 0.05; males, median 3.70 g, range 0.89–4.90 g, *n* = 5; females, median 1.10 g, range 0.58–3.09 g, *n* = 8).

**FIGURE 8:**
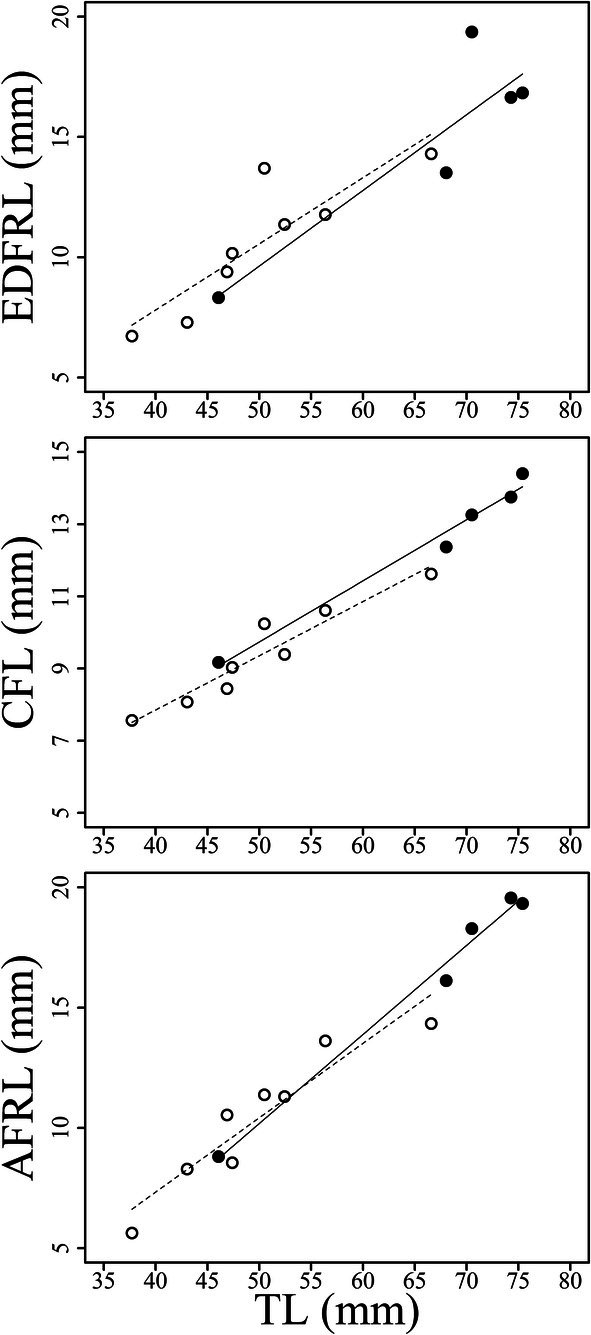
Relationship between total length (TL, mm) and elongated dorsal fin ray length (EDFRL, mm; top), caudal fin length (CFL, mm; middle) and anal fin ray length (AFRL, mm; bottom) in *Fusigobius inframaculatus*. Solid circles, males; open circles, females. The solid line indicates the linear regression for males (top, *y* = 0.31*x* − 6.09; middle, *y* = 0.17*x* + 1.27; bottom, *y* = 0.37*x* − 8.32). The dashed line represents the linear regression for females (top, *y* = 0.27*x* − 3.19; middle, *y* = 0.15*x* + 1.84; bottom, *y* = 0.31*x* − 5.03).

The ratio of elongated dorsal fin ray length (EDFRL) to TL was a median of 0.22 in males (range 0.18–0.27, *n* = 5) and 0.21 in females (range 0.17–0.23, *n* = 8). The regression line of EDFRL against TL did not significantly differ between sexes in either slope (ANCOVA, *t* = 0.38, *p* = 0.71) or intercept (ANCOVA, *t* = −0.47, *p* = 0.65). In the pooled data, TL was significantly and positively related to EDFRL (estimate ± SE = 0.30 ± 0.05, ANCOVA, *t* = 5.9, *p* < 0.001; Figure 8).

The ratio of caudal fin length (CFL) to TL was a median of 0.19 in males (range 0.18–0.22, *n* = 5) and 0.18 in females (range 0.17–0.19, *n* = 8). The regression line of CFL against TL did not differ significantly between sexes in slope (ANCOVA, *t* = 0.78, *p* = 0.46) or intercept (ANCOVA, *t* = 1.77, *p* = 0.11). In the pooled data, CFL was significantly and positively related to TL (ANCOVA, estimate ± SE = 0.16 ± 0.01, *t* = 13.3, *p* < 0.001; Figure 8).

The ratio of anal fin ray length (AFRL) to TL was a median of 0.26 in males (range 0.19–0.26, *n* = 5) and 0.22 in females (range 0.15–0.24, *n* = 8). The regression line of AFRL against TL did not differ significantly between the sexes in slope (ANCOVA, *t* = 1.08, *p* = 0.31) or intercept (ANCOVA, *t* = 0.37, *p* = 0.72). In the pooled data, AFRL was significantly and positively related to TL (ANCOVA, estimate ± SE = 0.34 ± 0.03, *t* = 11.9, *p* < 0.001; Figure 8).

## 4 DISCUSSION

The mating system of a fish species can be defined by the spatial distribution of males and females, and the mating relationships within local groups (Kuwamura, 1997; Sakai, 2023). In *F. inframaculatus*, males maintained non‐overlapping home ranges (i.e. territories) that included spawning nests, and territorial males were consistently the largest individuals within all study sites. Overlap in home ranges between males and females was generally low in Group 3. Thus, the mating system of *F. inframaculatus* corresponds to MTV polygamy (Kuwamura, 1997; Sakai, 2023).

Generally, mating relationships are unstable in MTV polygamy because males do not directly defend females (Kuwamura, 1997; Sakai, 2023). Females of *F. inframaculatus* were observed to change mates at sites where multiple males were present (e.g. Sites 2 and 3). However, females had no opportunity to change mates at sites where only a single male was present (e.g. Site 4). Many gobiid fishes are habitat specialists that occupy a variety of benthic microhabitats, such as sand, rocks and corals (Herler et al., 2011). *F. inframaculatus* inhabited shaded sandy bottoms within caves and beneath overhangs, using rocky crevices as shelters. The home range of this species was limited to shaded areas associated with rocky substrates. Shelter distribution is known to constrain migration and home range distribution in reef fishes (Karino et al., 2000; Sakai & Kohda, 1997; Sunobe & Nakazono, 1990). Variations in the mating relationships of this goby are likely linked to the shape and spatial distribution of rocky substrates that function as shelters.

Because intergroup migration or adult recruitment was rarely detected in *F. inframaculatus*, this species appears to have low mobility after settlement. This interpretation is further supported by the observation that all overwintering individuals remained at the same sites. As their home ranges were restricted to sandy bottoms within caves and overhangs, and seldom extended into rocky substrates, the configuration of rocky substrates likely limits intergroup movement. In addition, high predation risk may further constrain the active movement of females among their mates (Karino et al., 2000; Munday, 2002; Nakashima et al., 1995). The disappearance of 32% of the identified individuals within 4 months and nearly all (88%) by the next reproductive season suggests high predation pressure, which may further restrict movement between sites, therefore female mate availability is likely to be constrained by this limited mobility.

At Site 3, the home ranges of *F. inframaculatus* were linearly arranged along the edge of the rocky reef, with each individual adjacent to the next (Figure 7). This pattern likely reflects the continuous structure of rocky caves and overhangs that function as shelters. In the harem polygynous goby *Trimma okinawae* (Aoyagi, 1949), intraspecific variation in the spatial distribution of males and females has also been attributed to the morphology and distribution of shelters on rocky reefs (Sunobe & Nakazono, 1990). Two types have been described in *T. okinawae*; the hole type, where female home ranges are contained within the males' territory, and the cave type, where female home ranges are dispersed outside the male's territory. Similarly, in *F. inframaculatus*, harem‐like mating groups occurred at Site 4 under habitat conditions characterized by narrow cracks forming a communal shelter structure, where only a single male was present (Figure 7). Thus, in reef gobies associated with rocky environments involving *F. inframaculatus*, variation in spatial distribution likely arises from the size, shape and distribution of rocky substrates, and this spatial influence extends to mate availability.

In MTV polygamous species, particularly in Labridae and Scaridae, males often display conspicuous ornaments or bright body coloration (Kuwamura et al., 2000; Robertson & Hoffman, 1977; Warner & Schultz, 1992). These bright morphological traits in males are considered to have evolved primarily through female choice (Kuwamura et al., 2000; Warner & Schultz, 1992). Randall (1994) reported longer dorsal‐fin rays in male *F. inframaculatus* based on limited samples (four males and two females). However, in the present study, no conspicuous sexual dimorphism, other than larger body size and temporal body colour change, was detected in male *F. inframaculatus* (Figure 8). Cryptobenthic fishes are thought to rely primarily on substrate‐associated crypsis to avoid detection by predators (Brandl et al., 2018; Mihalitsis et al., 2024), and *F. inframaculatus* exhibits a camouflage coloration that matches sandy substrates. In this species, which is exposed to high predation pressure, conspicuous morphology may be disadvantageous for its survival. In the present study, males were found to exhibit a change in body coloration only during courtship. Such cryptic sexual dimorphism may play an important role in female choice in cryptobenthic fishes. In addition, sexual size dimorphism is associated with male–male competition for spawning nests (Hernaman & Munday, 2007). In the case of *F. inframaculatus*, any individuals within a social group, including females, are potential competitors for spawning nests due to hermaphroditic sexuality. Therefore, males of this goby are likely required to establish dominance within social groups to maintain their reproductive status as nest‐holding males. A large body size is widely recognized as an important factor in establishing dominance in males of protogynous fishes (Sakai, 2023) and this is also likely the case in *F. inframaculatus*.

In gobiid fishes, monogamy and harem polygyny are typically found in species inhabiting closed habitats such as coral patches, empty shells and cracks or holes in rocky substrates (e.g. *Paragobiodon*, Kuwamura et al., 1993; *Gobiodon*, Nakashima et al., 1996; *Lubricogobius*, Oyama et al., 2025; *Trimma*, *Priolepis*, Sunobe & Nakazono, 1990, 1999). In contrast, MTV polygamy occurs in species that use open habitats, such as sandy bottoms or exposed rocky surfaces, including *F. inframaculatus* (e.g. *Hazeus*, Kawase & Tsuhako, 2026; *Asterropteryx*, Manabe et al., 2009; *Bathygobius*, Taru et al., 2002; *Fusigobius*, Tsuboi & Sakai, 2016). These patterns suggest that extensive open habitats, which facilitate the formation of large social groups, are key to the evolution of MTV polygamy in gobiid fishes.

In *F. inframaculatus*, MTV polygamous mating groups were demonstrated (Sites 2 and 4), with variations involving facultative harem polygyny (Site 3), corresponding to habitat‐specific conditions. The narrow sheltering habitats with the low mobility of the goby, which may potentially constrain mate availability, do not necessarily preclude the establishment of an MTV polygamous group. Individual density is known to influence the establishment of mating systems in some gobiid fishes (Hernaman & Munday, 2007). Similarly, the aggregated distribution pattern of adult individuals observed in the study population may be associated with the establishment of a polygamous mating system in *F. inframaculatus*. Settlement patterns of juveniles as a key factor determining the individual distribution of this goby are expected to be elucidated in the future.

In a congeneric *F. neophytus*, sneaking tactics by small floating males have been reported, suggesting the coexistence of different life‐history pathways (diandry, i.e. primary and secondary males) within the species (Tsuboi & Sakai, 2016). In the present study, sneaking was not observed in *F. inframaculatus*; however, a floating male was observed. This observation indicates that a diandric life‐history pattern may exist in this species. The tactical advantages and life‐history pathways of floating males in *Fusigobius* require further investigation.

The genus *Fusigobius* is a hermaphroditic gobiid lineage that exhibits protogyny (Burns & Cole, 2017; Cole, 2010). The association between the MTV polygamous mating system and protogynous sexuality in *F. inframaculatus* is consistent with the predictions of the SA model (Warner et al., 1975). Dominant individuals within each group of *F. inframaculatus* were always males (Table S1), consistent with the species' protogynous sexuality (Burns & Cole, 2017). Males had higher reproductive success than females (Figure 5), supporting the adaptive significance of protogyny (Kuwamura et al., 2020; Sakai, 2023; Warner, 1984). In polygynous mating systems, protogynous sex change often occurs according to dominance hierarchies related to body size (Sakai, 2023). Given the observed male aggression toward females, a similar dominance‐based sexual pattern is likely to occur in *F. inframaculatus*. Further research on this topic is required.

## AUTHOR CONTRIBUTIONS

R.S.: Conceptualization, investigation, formal analysis, data curation, funding acquisition, writing – original draft preparation, writing – review and editing. Y.S.: Conceptualization, formal analysis, supervision, resources, writing – original draft preparation, writing – review and editing.

## FUNDING INFORMATION

This work was supported by JST SPRING (Grant Number JPMJSP2132) to Ryoga Seiwa.

## CONFLICT OF INTEREST STATEMENT

The authors declare that they have no conflicts of interest.

## ETHICS STATEMENT

All procedures complied with the current laws of Japan and were conducted in accordance with the Guidelines for the Proper Conduct of Animal Experiments outlined by the Hiroshima University Animal Research Committee, and the Guidelines for the Use of Fishes in Research by the Ichthyological Society of Japan (https://www.fish-isj.jp/english/journal/guidline/).

## Supporting information


**TABLE S1.** List of individuals of *Fusigobius inframaculatus* at the start of the study on the reefs of Kuchierabu‐jima Island. Changes in individual status, that is, disappearance (the last observation day) or sex changes (the first mating day as males) by the end of the survey (September 2024), are noted.

**DATA S1.** Seiwa and Sakai Video S1.mp4. The video shows the courtship and spawning behaviour of the *Fusigobius inframaculatus*.

## Data Availability

Data are available from Ryoga Seiwa upon reasonable request.
