# Supplementary material for: Male‐territory‐visiting polygamy in the sand‐dwelling goby Fusigobius inframaculatus (Gobiidae) inhabiting reef caves
Source: J Fish Biol. 2026 Mar 24;109(1):337–47. doi: 10.1111/jfb.70422 (PMC13397156; doi:10.1111/jfb.70422)
Supplement: Supplementary file 1 — TABLE S1. List of individuals of Fusigobius inframaculatus at the start of the study on the reefs of Kuchierabu‐jima Island. Changes in individual status, that is, disappearance (the last observation day) or sex changes (the first mating day as males) by the end of the survey (September 2024), are noted. [file JFB-109-337-s001.docx]

**Male-territory-visiting polygamy in the sand-dwelling goby *Fusigobius inframaculatus* (Gobiidae) inhabiting reef caves**

**Supporting information**

**Table S1.** List of individuals of *Fusigobius inframaculatus* at the start of the study on the reefs of Kuchierabu-jima Island. Changes in individual status, that is, disappearance (the last observation day) or sex changes (the first mating day as males) by the end of the survey (September 2024), are noted.

|  |  |  |  | Subsequent changes of individual status | | |
| --- | --- | --- | --- | --- | --- | --- |
| Site | ID | Sex | TL (mm) | Date  of disappearance | Sex change  to male (new ID) | Survived to 2025 |
| Site 1 | M15 | Male | 67 | June 24 |  |  |
|  | F1 | Female | 62 |  | July 1(M1) | Yes |
|  | F31 | Female | 48 | August 26 |  |  |
| Site 2 | M32 | Male | 75 |  |  |  |
|  | M2 | Male | 73 | August 5 |  |  |
|  | M18 | Male | 66 | June 29 |  |  |
|  | F29 | Female | 62 |  |  |  |
|  | F24 | Female | 61 |  | August 18 (M24) |  |
|  | F5 | Female | 58 |  |  |  |
|  | F33 | Female | 57 |  |  | Yes |
|  | F36 | Female | 57 |  |  |  |
|  | F6 | Female | 53 |  |  |  |
|  | F4 | Female | 46 | August 6 |  |  |
|  | F34 | Female | 45 | August 25 |  |  |
|  | F16 | Female | 44 |  |  |  |
|  | F7 | Female | 41 |  |  |  |
| Site 3 | M8 | Male | 71 |  |  |  |
|  | M27 | Male | 68 |  |  |  |
|  | F13 | Female | 66 | August 26 |  |  |
|  | F23 | Female | 63 |  | September 22 (M23) |  |
|  | F21 | Female | 62 |  |  |  |
|  | F17 | Female | 60 |  |  |  |
|  | F35 | Female | 60 |  |  |  |
|  | F26 | Female | 59 |  |  | Yes |
|  | F19 | Female | 58 |  |  |  |
|  | F9 | Female | 57 |  |  |  |
|  | F30 | Female | 57 | July 31 |  |  |
|  | F37 | Female | 56 |  |  |  |
|  | F20 | Female | 53 | July 9 |  |  |
|  | F28 | Female | 51 | August 12 |  |  |
|  | F14 | Female | 49 |  |  |  |
| Site 4 | M11 | Male | 73 |  |  |  |
|  | F22 | Female | 70 |  |  |  |
|  | F10 | Female | 68 | July 5 |  |  |
|  | F39 | Female | 52 |  |  |  |
|  | F12 | Female | 51 |  |  |  |
|  | F25 | Female | 50 |  |  |  |
